# Pediatric AIDS in the Elimination Agenda

**DOI:** 10.1371/journal.pmed.1001503

**Published:** 2013-08-27

**Authors:** Scott E. Kellerman, Nandita Sugandhi

**Affiliations:** 1 Management Sciences for Health, Arlington, Virginia; 2 Clinton Health Access Initiative, New York, New York

## Abstract

Scott Kellerman and colleagues argue that the scope of the current HIV elimination agenda must be broadened in order to ensure access to care and treatment for all children living with HIV.

*Please see later in the article for the Editors' Summary*

**Summary Points**

- Despite the global initiative to eliminate mother-to-child transmission of HIV, 210,000 new pediatric infections were added worldwide in 2012 to the existing pool of 3.4 million children living with the virus.
- Children are more vulnerable to HIV infection and have higher morbidity and mortality. Without treatment, one half of those children infected will die before the age of 2 years, yet only one third of those eligible for treatment are currently receiving antiretroviral therapy.
- Current initiatives focus on interventions within the traditional prevention of mother-to-child transmission cascade, but the scope of the elimination agenda must be broadened in order to ensure access to care and treatment for all children living with HIV.

## The Global Plan for Elimination of Pediatric HIV

In 2011, Ambassador Eric Goosby of the U.S. President's Emergency Plan for AIDS Relief (PEPFAR) and Michel Sidibe, Executive Director of Joint United Nations Programme on HIV and AIDS (UNAIDS), formally announced a plan for eliminating new HIV infections in children and keeping their mothers alive [1]. The elimination of pediatric HIV agenda, or the Global Plan, calls for decreasing new pediatric infections by 90% and halving maternal deaths from HIV and AIDS by 2015 [1]. This newest call to action strengthens previous global commitments to reduce the number of vertical HIV infections with concomitant decreases in mortality from HIV and AIDS and mortality in children under age 5 [2]. While high-level rhetoric is necessary to mobilize resources, the strategy to end mother-to-child transmission of HIV (eMTCT) has thus far focused primarily on the expansion of prevention of mother-to-child transmission (PMTCT) [1]–[3] with little attention focused on infected children or those missed by current programming. This strategy places at risk a whole generation of children who despite our best efforts are missed by current PMTCT programming and continue to become infected with HIV.

Eliminating MTCT is a worthy aspiration, and while the 2015 goal is ambitious, we are closing the gap. Expanding the focus on pediatric HIV is a collective effort and many experts in the field are aligned on what they know to be the existing barriers and potential solutions to ensuring equity in access to care and treatment for children infected and affected by HIV. The evolution of the WHO treatment guidelines is a case in point. The 2010 WHO PMTCT guidelines called for earlier initiation and extended periods of prophylaxis in pregnant and breastfeeding women to ensure protection throughout the duration of potential HIV exposure. Consensus has since emerged that earlier introduction of antiretroviral therapy (ART) in pregnant women is safer than imagined and results in lowered viral load and decreased vertical transmission [4]. The 2013 WHO guidelines will move us closer to a “test and treat” paradigm by expanding recommendations for lifelong ART for all pregnant and lactating women [5], which is routine practice in developed countries where vertical transmission is now seen as a rare but tragic curiosity [6].

## Challenges in Eliminating Pediatric AIDS

The goal of eliminating pediatric AIDS will elude us, however, if we continue to rely solely on PMTCT scale-up [7]. In the 2 years since the announcement of the Global Plan, the number of new infections has decreased by only 38% from 2009 levels in the 21 priority countries where 90% of HIV-positive pregnant women reside [8]. Challenges that remain in these countries include low antenatal clinic attendance and retention in care, suboptimal adherence to therapy, and non-existent case finding measures to identify HIV-infected children [9].

Eliminating pediatric AIDS requires developing and expanding strategies to reach children missed by current programming. We know that early treatment for HIV-positive children offers their best hope for survival [10],[11], and we have the ability to diagnose and deliver such treatment. Despite impressive scale-up of PMTCT programming in high-burden countries such as Botswana and Namibia, and the implementation of Option B+ in Malawi [12], Uganda, and elsewhere, vertical transmission and pediatric HIV infection remain significant challenges throughout the world. Countries such as the Democratic Republic of Congo and Nigeria are falling behind efforts to eliminate new infections and contribute nearly half of the global burden of new pediatric infections [8]. In 2012, 210,000 new pediatric infections were added to the pool of 3.4 million children infected with HIV worldwide, and while data from 2012 are not yet available, in 2011, 230,000 children died from AIDS-related illnesses [8].

Although slowly improving, ART coverage for more than 2 million children living with HIV [13] is dismally low despite clear evidence that early treatment for HIV-infected children can keep children alive to adulthood [10],[14]. At the end of 2012, 76% of children eligible to receive ART in the 21 priority countries were not being treated compared to 35% of eligible adults [8]. While much work has focused on identifying gaps in PMTCT access, far less has focused on reducing gaps for linking and retaining infected children in care.

Even within PMTCT programs, a primary emphasis on nucleic acid testing at 6 weeks of life only identifies those children infected in utero or during delivery. While minimized with the introduction of extended prophylaxis, the risk of transmission through breastfeeding continues for up to 2 years, and few exposed infants are retested during this period [15]. Retesting of breastfeeding mothers and their male partners is particularly urgent in high-incidence settings where acute infections may be driving new pediatric infections [16],[17]. Repeat testing in previously negative pregnant women at delivery has been shown to be cost effective [15], and given the duration of breastfeeding, savings could be significant if retesting was considered for the duration of the exposure period.

## Expanding the Agenda to Include All Children

We must do a better job of identifying HIV-infected children missed by current PMTCT programming. The infants of mothers who never enter or complete PMTCT are those most at risk for infection. These infants are not the ones who arrive at maternal-child clinics for their 6-week dried-blood spot test, but are the children of mothers who never received antenatal care, were never tested during pregnancy, seroconverted after testing, delivered at home, were non-adherent to therapy, or were lost to follow-up. Early infant diagnosis relies on retention of the mother-infant pair in PMTCT but is not designed to find children who were never enrolled. In sub-Saharan Africa, home to 92% of pregnant women living with HIV, 59% of HIV-infected pregnant women received antiretroviral therapy or prophylaxis in 2011. This is a fantastic achievement but it only serves to underscore that more than 40% of infants remain vulnerable. Waiting until these HIV-infected children become sick and present late for care and treatment has proven to be an inadequate strategy [18].

Sensitization of healthcare workers to the urgency of early identification and treatment is a critical gap. Finding infected and at-risk children will require HIV screening and/or testing at immunization and sick-child clinics in high-prevalence settings and at all pediatric hospital admissions. The Integrated Management of Childhood Illness (IMCI) can be an effective tool for identifying HIV-infected and exposed children but is not used routinely by health workers, and incorporation of HIV identification into childhood clinic visits remains scattered and underutilized [19]. The expansion of family-centered testing using parents as index cases may further identify previously missed, HIV-infected children when they are still healthy. These approaches are often discussed but remain uncommon in practice and deserve evaluation so that more effective case-finding can be implemented.

Once HIV has been diagnosed, treatment should be easily and rapidly accessible, with further decentralization of pediatric HIV care, utilization of child-friendly antiretroviral drug formulations, and simplified treatment regimens. The use of cumbersome syrups and carefully calibrated dosing once relegated pediatric treatment to the realm of specialized care. Phasing out such formulations in favor of fixed-dose combination tablets streamlines treatment protocols. Even so, pediatric-specific challenges to adherence and retention in care need to be addressed as dependency on an adult caregiver, lack of disclosure, and HIV-infected children maturing to adolescence all pose unique challenges to effective pediatric treatment. If we can overcome the barriers to early initiation of children on ART, we can expect that those children will survive to adolescence, necessitating the creation of adolescent friendly programs to address new challenges around sexual debut and transmission and transitioning to adult care.

The benefits of early treatment must be recognized by caregivers and communities as well. Appropriate messaging about PMTCT and pediatric case-finding can successfully mobilize communities and bring mothers into care, although guilt, shame, and hopelessness often prevail [20]. How can we better stress the benefits of early treatment for infected children to dispel beliefs that an infected child is a lost cause?

The increased focus on eMTCT is welcome, but it is not enough. A new and more expansive agenda must be articulated to ensure its effects reach those infants and children who will never feel the impact of the current elimination agenda. This expanded agenda must addresses challenges around reducing vertical transmission and ensuring access to appropriate HIV testing, care, and treatment for all affected children.

## Abbreviations

ART: antiretroviral therapy
eMTCT: ending mother-to-child transmission of HIV
PMTCT: prevention of mother-to-child transmission
